# Beyond Glycaemic Thresholds: Towards a Comprehensive Metabolic Understanding of Gestational Diabetes

**DOI:** 10.3390/nu18081295

**Published:** 2026-04-20

**Authors:** Sarah Stromberger, Andrea Tura, Christian Göbl

**Affiliations:** 1 Division of Obstetrics and Feto-Maternal Medicine, Department of Obstetrics and Gynaecology, Medical University of Vienna, 1090 Vienna, Austria; sarah.stromberger@meduniwien.ac.at; 2 CNR Institute of Neuroscience, 35127 Padova, Italy; andrea.tura@cnr.it

Gestational diabetes mellitus (GDM) is increasingly recognised as a condition not defined by glucose thresholds alone, but as a manifestation of broader metabolic dysregulation. The rising prevalence of hyperglycaemia in pregnancy reflects the global burden of obesity and underlying metabolic disorders, underscoring the need to reconsider maternal metabolism across pregnancy and beyond [1,2,3]. Although glucose remains central for the diagnosis and management of GDM, a glucose-centred framework captures only part of the complex and heterogeneous metabolic mechanisms.

Even physiological pregnancy represents a dynamic metabolic state characterised by progressive insulin resistance, adaptive hormonal changes, and a shifting of substrate utilisation [3]. Hyperglycaemia emerges when compensatory insulin secretion fails to match this demand. However, this paradigm alone does not adequately explain the marked variability in clinical presentation and outcomes observed in GDM (contribution 1) [3,4].

Increasing evidence indicates that adverse pregnancy outcomes cannot be attributed to glucose alone, but rather reflect a broader metabolic milieu. This becomes particularly evident in the context of foetal growth. While maternal glycaemia contributes to foetal overgrowth, additional pathways, including inflammatory signalling, placental regulation, and growth factor activity, could potentially play further critical roles. As highlighted in this Special Issue, aberrant cytokine signalling may represent a key mechanism linking maternal metabolism to foetal macrosomia (contribution 2). Importantly, clinical data further support this broader perspective. In a large retrospective cohort, perinatal outcomes in women with GDM were not primarily determined by the method of glycaemic management, but were rather influenced by age, mode of delivery, and maternal comorbidities (contribution 3). These observations challenge the notion that glycaemic control alone is sufficient to mitigate risks for the growing foetus.

Lifestyle interventions, specifically medical nutrition therapy (MNT), remain the cornerstone of GDM management; however, defining optimal dietary strategies continues to be challenging. As reviewed by Cheong and coauthors, substantial gaps persist between metabolic understanding and effective nutritional implementation (contribution 4): beyond carbohydrate intake, the quality and composition of macronutrients, as well as micronutrient status, influence insulin sensitivity, inflammatory processes, and foetal development. However, the translation of these insights into clinical practice is limited. Sociocultural, behavioural, and economic factors strongly affect dietary adherence, highlighting the disconnection between theoretical recommendations and real-world applicability (contribution 4). These findings emphasise that looking beyond a uniform nutritional approach is essential to adequately address a wide range of factors influencing the successful treatment of GDM.

Beyond nutrition, increasing attention has been directed towards metabolic conditions that coexist with or precede GDM. Metabolic dysfunction-associated fatty liver disease (MAFLD), now surpassed by the definition of metabolic dysfunction-associated steatotic liver disease (MASLD) [5], represents a hepatic manifestation of systemic metabolic imbalance with important implications for pregnancy (contribution 5). Evidence suggests a bidirectional relationship, whereby hepatic steatosis in early pregnancy increases the risk of subsequent GDM, while GDM predisposes to postpartum MASLD. Both conditions are associated with adverse pregnancy outcomes and long-term cardiometabolic risk, reinforcing the concept of pregnancy as a critical window for the identification of metabolic vulnerability (contribution 5).

In parallel, skeletal muscle (despite being the primary site of insulin-mediated glucose disposal) remains largely overlooked in the context of pregnancy. Sarcopenia, characterised by reduced muscle mass and function, shares key pathophysiological mechanisms with type 2 diabetes, including insulin resistance, chronic inflammation, and altered energy metabolism (contribution 6). Given that pregnancy is intrinsically a state of physiological insulin resistance, these mechanisms may be amplified, potentially influencing both maternal glucose homeostasis and foetal growth. Yet, data on the prevalence and clinical relevance of sarcopenia in pregnancy remain limited, underscoring a critical gap in current metabolic frameworks (contribution 6) [6].

Taken together, the contributions within this Special Issue support a shift from a glucose-centred definition of GDM towards a multidimensional metabolic model. Hepatic and skeletal muscle alterations, inflammatory pathways, and placental signalling emerge as interconnected components of a complex metabolic network. This perspective provides a more coherent explanation for the heterogeneity of GDM and highlights the limitations of current diagnostic and therapeutic strategies that rely predominantly on glycaemic thresholds.

Future research should move beyond hyperglycaemia and aim to integrate these metabolic dimensions into refined phenotypic classifications. Identification of high-risk metabolic profiles, ideally in early pregnancy, may enable more targeted and effective interventions. In parallel, longitudinal approaches extending beyond pregnancy are essential to capture long-term cardiometabolic risk in both mothers and offspring. Ultimately, advancing from a glucose-centred paradigm to a comprehensive metabolic understanding of GDM represents a necessary step towards truly individualised, pathophysiology-driven care.
